# Intermittent and episode-driven use of pranlukast to reduce the frequency of wheezing in atopic children: a randomized, double-blind, placebo-controlled trial

**DOI:** 10.1186/s40413-015-0062-3

**Published:** 2015-04-02

**Authors:** Motohiro Ebisawa, Akihiko Terada, Kazuki Sato, Fumitake Kurosaka, Naomi Kondo, Chizuko Sugizaki, Akihiro Morikawa, Sankei Nishima, Mitsuyoshi Urashima

**Affiliations:** Department of Allergy, Clinical Research Center for Allergy and Rheumatology, Sagamihara National Hospital, Sagamihara, Japan; Department of Pediatrics, Daido Hospital, Nagoya, Japan; Department of Pediatrics, National Shimoshizu Hospital, Yotsukaido, Japan; Kurosaka Pediatrics and Allergy Clinic, Himji, Japan; Gifu University Graduate School of Medicine, Gifu, Japan; Gunma University & Kitakanto Allergy Institute, Maebashi, Japan; Allergy division, Fukuoka National Hospital, Fukuoka, Japan; Division of Molecular Epidemiology, Jikei University School of Medicine, Nishi-shimbashi 3-25-8, Minato-ku, Tokyo 105-8461 Japan

**Keywords:** Pranlukast, Leukotriene receptor antagonist, Asthma exacerbations, Japan, Children

## Abstract

**Background:**

Leukotriene receptor antagonist (LTRA) therapy reduces asthma exacerbations in children older than 2 years. However, whether early intervention using LTRA in atopic smaller children aged 1 to 2 years who had experienced episodic wheezing can reduce the frequency of wheezing is unknown.

**Methods:**

A randomized, double-blind, placebo-controlled, multi-center trial of episode-driven intermittent use of pranlukast for 12 months, one of the LTRAs, was conducted by enrolling children who had two, but not more than two, episodes of wheezing prior to entry and were allergen-specific IgE-positive (≥class 2). The primary outcome was increased episodes of wheezing more than once a month for 3 months.

**Results:**

Seventy-seven children were randomly assigned to receive pranlukast (n = 37) or placebo (n = 40). The primary outcome occurred in 10 of 36 (28%) of the pranlukast group and 14 of 39 (36%) in the placebo group, which was not significantly different (P = 0.45). Even though the study period was extended to a maximum of >5 years, there was no significant difference in the Kaplan-Meier curves in the occurrence of the primary outcome between the two groups.

**Conclusions:**

These results suggest that intermittent and episode-driven use of pranlukast in small children with a prior history of wheezing and atopic sensitization may not reduce the frequency of wheezing later in life. However, the sample size was too small to make a definitive conclusion.

**Trial registration:**

UMIN000000634

## Introduction

Children infected with respiratory syncytial virus in early life are at increased risk of developing asthma during the school years [1]. Upper respiratory viral infections are also associated with 80-85% of asthma exacerbations in school-age children [2]. Of interest, virus-induced asthma attacks occurred less in children on inhaled corticosteroid and/or leukotriene receptor antagonist (LTRA) therapy in a case–control study [3]. In addition, montelukast, one of the LTRAs, was shown to reduce asthma exacerbations in children with intermittent asthma, which is sometimes very difficult to differentiate from viral infections, especially in preschool children, with over 12 months of treatment in the PREVIA study (2- to 5-year-olds) [4] and the PRE-EMPT study (2- to 14-year-olds) [5]. On the other hand, atopic children have been shown to experience more colds and asthma exacerbations than non-atopic children [6]. However, whether early intervention using an LTRA in atopic smaller children aged 1 to 2 years who had experienced episodic wheezing but were not yet diagnosed as having asthma can reduce the frequency of wheezing episodes was unclear. Therefore, a randomized, double-blind, placebo-controlled trial was conducted to determine whether intermittent and episode-driven use of pranlukast, one of the LTRAs, could improve control of wheezing symptoms in small children aged 1 to 2 years with atopic sensitization and two episodes of wheezing prior to entry.

## Methods

### Trial design

This trial was a balanced, randomized [1:1], double-blind, placebo-controlled, parallel-group study conducted at multiple medical institutions. Participants were enrolled from April 2007 to April 2010. The trial protocol was developed by all authors and approved by the ethics committee or the institutional review board of Sagamihara National Hospital, the Jikei University School of Medicine, Chiba University Graduate School of Medicine, Gifu University Graduate School of Medicine, Saiseikai Nakatsu Hospital, Fukuoka National Hospital, Shimoshizu National Hospital, Saga University, Niigata Prefecture Yoshida Hospital, Yamaguchi University Graduate School of Medicine, Dokkyo University, Miyagi Children's Hospital, Tsukuba Medical Center Hospital, Gunma Children's Medical Center, Toho University Ohashi Medical Center, Daido Hospital, Toyama Red Cross Hospital, Moriyama Municipal Hospital, Kochi University, Showa University, Ebara Hospital and Sado General Hospital. Since private clinics do not have their own institutional review boards, the approval by the ethics committee of Sagamihara National Hospital was applied for these private clinics: Nishioka Allergy Clinic, Nakano Children's Clinic, Watanabe Pediatrics & Allergy Clinic, Watanabe Pediatric Clinic, Shichijo Pediatric Clinic, Kurosaka Pediatrics & Allergy Clinic, Kawahara Pediatrics & Allergy Clinic, Tsubaki Children's Clinic, Uekusa Pediatric Clinic, Shimizu Pediatrics & Allergy Clinic, and Eniwa Daiichi Hospital. The trial (number UMIN000000634) was registered with UMIN Clinical Trials Registry on April 1, 2007. The data monitoring center was at the Division of Molecular Epidemiology, the Jikei University School of Medicine. Both pranlukast and placebo were provided by Ono Pharmaceutical Co., Ltd. (Osaka, Japan), which had no control of protocol development, randomization, blinding, data monitoring, statistical analysis, or manuscript writing. Written, informed consent was obtained from the guardians of participants on behalf of their infants enrolled in this study.

### Study population, eligibility, and consent

Inclusion criteria were infants aged 1 to 2 years at entry who had just two episodes of wheezing before entry and atopic sensitization to at least one allergen-specific IgE ≥ class 2. Excluded were infants: 1) who already had three or more wheezing episodes; 2) who had a history of taking pranlukast for more than 6 months; or 3) who had a history of take antihistamines for more than 6 months. Enrollment was done by the collaborating pediatricians who were blinded to allocated group. When children satisfied the inclusion criteria, the collaborating pediatricians explained the trial to the families at the outpatient clinic and asked them to participate in the trial.

### Randomization, blinding, and intervention

A central computer was used to randomly assign patients into permutated blocks of four to receive either pranlukast (total 80 mg/day) or placebo. The pranlukast and placebo were in powder form and identical in appearance and taste. These powders were pre-packaged in envelopes and a small box and consecutively numbered for each patient according to the randomization schedule. Randomization and the blinding process were performed by MU, who had no clinical involvement in this trial. After obtaining written, informed consent, the patients were assigned to groups, given the corresponding pre-packaged small box, and asked to take the powder two times per day for at least 2 weeks when the participating children had symptoms of a common cold (runny nose, cough, and/or wheezing). If these symptoms continued for more than 2 weeks, the trial medicine was administered to cover the whole symptomatic period. We call this method of administration “intermittent and episode-driven use”. This intermittent use of the study drugs was continued for 12 months.

### Follow-up procedures

The collaborating pediatricians checked patients’ characteristics at baseline and asked them to visit the outpatient clinic and see the same pediatrician once every 4 weeks. When symptoms appeared, the participants were asked to also visit the collaborating pediatricians. Co-interventions with a β_2_ agonist and antitussives/expectorants were allowed during these symptoms. At every 4-week visit, the collaborating pediatricians evaluated wheezing by physical examination, asked about the patient’s condition during the previous 4 weeks, and checked the Japanese asthma diary. Adherence to the trial medicine was evaluated based on the diary. In addition, after the intervention period, all trial medicine was collected, and the numbers of packages used and not used were counted for each participant.

### Outcome measure

The primary outcome was defined as increased frequency of wheezing: episodes of wheezing more than once per month, which continued for 3 months.

The secondary outcomes were frequency of major (severe wheezing with orthopnea, etc.), moderate (clear wheezing with hypoxia but without orthopnea etc.), and mild (wheezing without hypoxia) episodes of wheezing. In addition, the numbers of weeks of wheezing, of difficulty breathing, of heavy cough, and of light cough based on the Japanese asthma diary checked by the participant’s parent(s) were counted. Moreover, use of rescue medication (short-acting β_2_-agonists, inhaled glucocorticosteroids, and systemic use of glucocorticosteroids) was compared between the two groups.

All outcomes were measured by the collaborating pediatricians and locked by one of the investigators (ME); all were blinded to allocated group.

### Statistical analysis

It was estimated that the outcome would occur in 50% of infants in the placebo group within 1 year. An equally divided sample of 200 was calculated as being sufficient for the detection of a 40% reduction in outcome, with a type I error (two-sided) of 5% and a power of 80%, on the assumption of no loss to follow-up.

To compare patients’ characteristics between the groups, Student’s *t*-test and the Mann–Whitney test were used for continuous variables with normal and non-normal distributions, respectively. For binary variables, the χ^2^ test was used. To analyze the outcome measures, an intention-to-treat (ITT) analysis was used. The outcomes were compared between the pranlukast group and the placebo group, and the risk ratio (RR), risk difference (RD), and 95% confidence interval (95%CI) were determined. All reported *P* values are two-sided. *P* values < 0.05 were considered significant. All analyses were performed by MU who did not examine the patients. Stata 13.1 (StataCorp LP, College Station, TX) was used for all analyses.

During the 3 years from April 2007 to April 2010, attempts were made to enroll 200 participants, but only 77 were enrolled. Therefore, new entry was stopped on April 2010, but the study period was extended to October 2012 to obtain more primary outcomes. The asthma was further classified into intermittent, mild persistent (asthma attack < 1/month), moderate persistent (1/month ≤ asthma attack < 1/week), and severe (asthma attack almost every day), before starting inhaled corticosteroid and/or LTRA. These outcomes were confirmed by the collaborating pediatricians and locked by the investigator (ME) without knowing the allocated group of each participant. Asthma-free rates were compared by creating Kaplan-Meier curves and using the log-rank test.

## Results

### Study population

**Figure 1:**
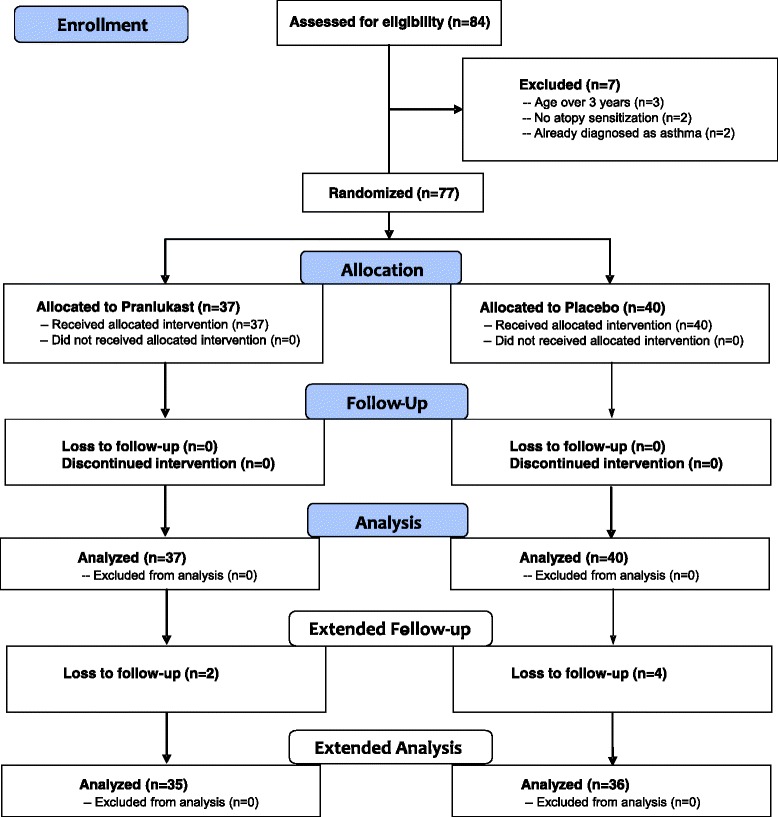
**Flow diagram of pranlukast versus placebo.** The diagram includes detailed information on the excluded participants.

A flow diagram is shown in Figure 1. A total of 84 parents were asked to participate in this trial, and 77 infants were randomly assigned to receive pranlukast (n = 37) or placebo (n = 40) in a double-blind setting for 12 months from April 2007 to April 2010. Because it took more than 3 years to enroll only less than 40% of the planned sample size, it was decided to stop entry of new participants on April 2010. Instead, the study period for following the participants was extended as long as possible to October 2012, maintaining the double-blind. All participants were followed-up for the first year as planned, although two and four children were lost to follow-up in the pranlukast group and in the placebo group, respectively, during the extended study period. All participants were confirmed to have taken the trial medicine during symptomatic periods as scheduled.

### Participants’ characteristics

The mean age of the study population was 1.9 years, with more boys than girls. More than half had food allergies, and around 40% had atopic dermatitis. Total IgE and allergen-specific IgE levels were relatively high in this population, since infants who showed atopic sensitization (≥class 2 to at least one allergen-specific IgE) were included. However, patients’ characteristics were similar in the two groups (Table 1).

**Table 1:** **Patients’ characteristics**

	**Total (n = 77)**	**Pranlukast (n = 37)**	**Placebo (n = 40)**	**P value**
Age (y), mean (SD)	1.9 ± 0.6	1.8 ± 0.5	1.9 ± 0.6	0.71^*1^
Males, no. (%)	56 (73)	29 (78)	27 (68)	0.28^*2^
Comorbidity^*3^				
Food allergy, no. (%)	39 (51)	20 (54)	19 (48)	0.57^*2^
Atopic dermatitis, no. (%)	32 (42)	14 (38)	18 (45)	0.52^*1^
Total IgE (IU), median (25% – 75%)	214 (76 – 585)	224 (64 – 536)	204 (106 – 771)	0.77^*4^
Allergen-specific IgE (IU), median (25% – 75%)				
Mite	5.8 (0.4 – 49)	5.6 (0.4 – 48)	6.1 (0.3 – 49)	0.42^*4^
Egg	5.6 (1.1 – 26)	12 (0.9 – 29)	3.2 (1.3 – 15)	0.26^*4^
Milk	0.7 (0. 4 – 4.3)	1.3 (0.4 – 9.9)	0.7 (0.4 – 2.5)	0.34^*4^
Wheat	0.6 (0.3 – 3.1)	0.5 (0.3 – 3.5)	0.7 (0.3 – 2.2)	0.96^*4^
Eosinophils (%), median (25% – 75%)	3.6 (1.4 – 5.0)	4.0 (1.4 – 5.0)	3.3 (1.0 – 5.0)	0.71^*4^

^*1^P value was calculated by Student’s *t*-test. ^*2^P value was calculated by the χ^2^ test. ^*3^Comorbidity was diagnosed by collaborating pediatricians. ^*4^P value was calculated by the Mann–Whitney test.

### Outcomes over the 1^st^ year

The effects of pranlukast on the primary and secondary outcomes are shown in Table 2. Asthma occurred in 10 of 36 (28%) in the pranlukast group compared with 14 of 39 (36%) in the placebo group, which was not significantly different (*P* = 0.45), even after adjusting for sex (*P* = 0.44). One participant in the placebo group was admitted to hospital due to an asthma attack. The frequencies of major, moderate, and mild wheezing were also not different between the groups. Use of rescue medications was also the same between the groups. Moreover, the numbers of weeks of wheezing, of difficulty breathing, of heavy cough, and of light cough were also not different (data not shown). Subgroup analyses were not performed because the number of participants was too small to detect significant differences.

**Table 2:** **Effects of pranlukast on the primary and secondary outcomes during the 1** ^**st**^ **year**

	**Pranlukast N/total (%)**	**Placebo N/total (%)**	**RR** ^*1^ **(95% CI** ^*2^ **)**	**RD** ^*3^ **(95%CI** ^*2^ **)**	**P value**
Primary outcome^*4^	10/36 (28)	14/39 (36)	0.77 (0.39 to 1.52)	−0.08 (−0.29 to 0.13)	0.45
Secondary outcomes					
Major attack	2/34 (6)	2/38 (5)	1.12 (0.17 to 7.51)	0.01 (−0.10 to 0.11)	0.91
Moderate attack	1/34 (3)	4/38 (11)	0.28 (0.03 to 2.38)	−0.08 (−0.19 to 0.04)	0.21
Mild attack	8/34 (24)	9/38 (24)	0.99 (0.43 to 2.28)	−0.00 (−0.21 to 0.16)	0.82
Rescue medication use					
Rapid-acting inhaled β_2_-agonist	12/36 (33)	16/39 (41)	0.81 (0.45 to 1.47)	−0.08 (−0.29 to 0.14)	0.49
Systemic glucocorticosteroid	3/36 (0.8)	1/39 (0.3)	3.25 (0.35 to 29.8)	0.06 (−0.05 to 0.16)	0.27
Inhaled glucocorticosteroid	1/36 (0.3)	1/39 (0.3)	1.08 (0.07 to 16.7)	0.00 (−0.07 to 0.08)	0.95

^*1^RR: Relative risk, ^*2^RD: 95%CI: 95% confidence interval, ^*3^Risk difference, ^*4^The primary outcome was defined as an increased frequency of wheezing: episodes of wheezing more than once per month, which continued for 3 months.

### Extended study

**Figure 2:**
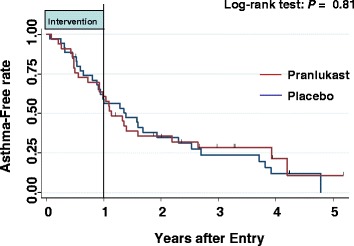
**Kaplan-Meier curves of the asthma-free rate.** The log-rank test was used to compare the curves of the pranlukast group and the placebo group.

Since enough participants could not be enrolled, the study period was extended for as long as possible to October 2012. As a result, the primary outcome was detected in 85% of the study population. However, there was no significant difference in the asthma-free rate between the pranlukast group and the placebo group, shown with the Kaplan-Meier curves (Figure 2). Moreover, there were no differences in inhaled corticosteroid use between the two groups (data not shown).

### Safety

During 1^st^ year of the study period, there were no reports of serious adverse events needing hospital admission, except one case of status asthmaticus.

## Discussion

LTRA has been reported to attenuate allergic airway inflammation in a mouse model sensitized with mite allergen and repeatedly infected with respiratory syncytial virus [7], which is similar to the pathophysiology of the virally-induced wheezing frequently observed in small children. Thus, this RCT was conducted. However, intermittent and episode-driven use of pranlukast did not reduce the frequency of wheezing in atopic and wheezy children under 3 years of age, not only for 12 months but also for longer. In another RCT (the AIMS trial) reported after starting this trial [8], the proportion of episode-free days during 12 months was demonstrated not to be different among inhaled corticosteroid, LTRA, and conventional therapy in pre-school children aged under 5 years with intermittent wheezing. More recently, montelukast was shown not to reduce the number of asthmatic episodes over 1 year in pre-school children [9]. These two recent RCTs are consistent with the results of the present study.

In contrast to the present results, the initial RCTs [4,5] that targeted the age group of preschool children ≥2 years old, who were already diagnosed as having intermittent asthma, showed significant effects of LTRA therapy, whereas recent RCTs and the present study, which evaluated toddlers ≥ 1 year old who had episodic wheezing but were not yet diagnosed as having genuine asthma, showed no significant effect of LTRA therapy. The effect of montelukast for children older than 2 years of age diagnosed as having persistent asthma has already been demonstrated [10]. In contrast, this trial evaluated the reduction in the frequency of wheezing, which is different from treatment for intermittent or persistent asthma. Moreover, these 4 previous major RCTs ran for 12 months; in contrast, the present maximum follow-up was extended for more than 5 years. In addition, the present trial targeted atopic small children, but the four previous RCTs did not always involve atopic patients. In this respect, the present study seems unique.

There are several limitations to this study. First, a sufficient number of participants to detect a difference could not be accrued. However, the study period was extended to more than five years. Second, only intermittent and episode-driven use of pranlukast was compared with placebo in this trial. Different regimens, including daily use, higher doses, or longer durations may lead to different findings. Third, wheezing of small children, especially under 3 years of age, can be associated with non-asthma diseases such as virus-induced bronchiolitis [11]. Thus, the study population of this trial included wheezing of heterogeneous etiologies. Fourth, episodic wheezing and cough are very common even in children who do not have asthma, particularly in those under the age of three years [12]. Thus, the outcome measure may be biased to include non-asthmatic wheezing.

## Conclusion

Intermittent and episode-driven use of pranlukast in small children with a prior history of wheezing and atopic sensitization may not reduce the frequency of wheezing later in life. However, the sample size was too small to make a definitive conclusion.
